# Infection prevention and control in daycare centers—a survey of caregivers’ knowledge and practices in Ogbomoso Community, Southwest Nigeria

**DOI:** 10.3389/fpubh.2025.1571710

**Published:** 2025-06-11

**Authors:** Rafiat Omotayo Ishola, Dolapo Emmanuel Ajala, Grace Olayanju, Deborah Oluwadamilola Ilugbaro, Ronke Gbonjubola Ajala, Taiwo Omotayo Dosumu, Eunice Oluwakemi Ogunmodede

**Affiliations:** ^1^Faculty of Nursing, College of Health Sciences, Bowen University, Iwo, Nigeria; ^2^Bowen University Hospital, Bowen University, Iwo, Nigeria; ^3^Clinical Nursing Department, Bowen University Teaching Hospital, Ogbomoso, Nigeria

**Keywords:** infection, prevention, control, daycare caregivers, Nigeria

## Abstract

**Introduction:**

Infectious diseases can spread rapidly among children in daycare centers (DCC). Caregivers at DCC play a crucial role in preventing and controlling these diseases.

**Objective:**

This study aimed to assess the knowledge and practice of infection prevention and control (IPC) among caregivers in DCC.

**Methods:**

A descriptive cross-sectional study was conducted involving daycare caregivers in the Ogbomoso community, Southwest Nigeria. The Taro Yamane formula was used to calculate a minimum sample size of 111 caregivers from a total population of 136. Due to the lack of a formal registry for daycare centers, the snowball sampling technique was employed; initial participants referred additional caregivers from their professional networks, which aided in recruiting this hard-to-reach population. A pretested, self-developed questionnaire, with a Cronbach’s alpha coefficient of 0.96, was administered. Data were analyzed using descriptive and inferential statistics (*α* = 0.05).

**Results:**

The findings from this study showed that the majority (60%) of the respondents were within the age group of 30–39 years. Overall, 40% of the respondents had good knowledge of IPC, while 47.3% exhibited poor practices related to IPC. Years of experience (*p* = 0.001) and knowledge (*p* = 0.000) were statistically significant with the practice of IPC.

**Conclusion:**

This study identified a significant association between caregivers’ knowledge and their practice of infection prevention and control (IPC), as well as between years of experience and IPC practice. We recommend implementing regular, targeted IPC training that specifically addresses the identified gaps in practice, particularly in hand hygiene and waste management, to further improve infection control standards in daycare centers.

## Introduction

Infection prevention and control (IPC) remains a fundamental aspect of public health, particularly in settings like daycare centers where young children, usually aged between 0 and 5 years, are highly vulnerable to infectious diseases (1). These settings, characterized by close personal interactions and shared activities, can quickly become hotspots for various infections, including those affecting the respiratory system, gastrointestinal tract, and skin (2, 3). Effective IPC strategies, which include appropriate hand hygiene, thorough environmental cleaning, and proper waste management, are crucial not only to reduce the transmission of infections but also to protect the health of children and caregivers in these settings (1, 4).

Although the significance of IPC is well recognized, there are growing concerns about its effective implementation in Nigerian daycare centers. Several caregivers, due to inadequate training and limited resources, do not fully adhere to the prescribed IPC guidelines, thereby creating an environment conducive to the spread of infectious agents (1, 5, 6).

Studies conducted in various regions of Nigeria have reported high rates of bacterial contamination and antibiotic resistance in daycare settings, highlighting the urgent need for improved infection control measures, better sanitation practices, and targeted public health interventions (7–9). In many rapidly urbanizing Nigerian cities, public health challenges such as overcrowding, inadequate sanitation, and limited access to healthcare are prevalent (10).

Ogbomoso North Local Government Area (LGA), the most populous region in the Ogbomoso community in Southwest Nigeria, presents a particularly relevant context for examining IPC in daycare centers. Rapid urbanization and commercialization have resulted in a surge of daycare facilities catering to working-class families; however, many of these centers face significant resource constraints and infrastructural challenges. In addition, reports of recurring infectious disease outbreaks in local childcare settings further emphasize the vulnerability of this population and the urgent need for effective IPC strategies.

Although the theoretical basis for IPC is well established in the broader healthcare literature, there is a notable lack of research that explores explicitly how these practices are implemented in daycare settings, particularly in low-resource circumstances (5, 11, 12). Previous studies have revealed alarmingly low levels of knowledge and inconsistent enforcement of IPC practices among caregivers, highlighting the urgent need for a detailed examination of these methods (1, 5, 6).

This study was designed to address these knowledge gaps by assessing the current status of IPC in terms of both knowledge levels and the practical measures taken by daycare caregivers in the Ogbomoso community. By systematically evaluating the actual conditions of IPC practices within these centers, the research aims to identify specific deficiencies, such as inadequate training and limited resources, that contribute to the insufficient prevention and control of infections (13). In doing so, the study not only addresses a significant gap in our understanding but also lays the groundwork for developing targeted interventions that can improve the effectiveness of IPC strategies in Nigerian daycare centers. Ultimately, this effort aims to enhance public health outcomes for the most vulnerable individuals in our communities (14).

## Materials and methods

### Study design

The study employed a cross-sectional descriptive design involving caregivers in DCC between February 2024 and April 2024. This design was selected to measure the knowledge and practices regarding infection prevention and control (IPC) among daycare workers in the Ogbomoso community. This approach allows for a “snapshot” of the present state of IPC practices among the target group at a specific point in time, offering a clear view of current strengths and weaknesses in caregivers’ behaviors. Given that earlier studies (5, 6) have reported considerable gaps in IPC awareness and inconsistent adherence to suggested protocols in similar settings, the cross-sectional descriptive method is particularly suitable for thoroughly identifying these concerns.

### Study setting

Ogbomoso North Local Government Area (LGA) is the largest in population among the five LGAs within the Ogbomoso community, situated in the northern part of Ogbomoso city, Oyo State, Southwest Nigeria. With an estimated population of approximately 298,410 as of 2025, this region is highly populated and has seen considerable urban development in recent years. Ogbomoso North covers approximately 183.6 to 207 km^2^ and is known for its robust level of commercialization, including numerous banks, markets, and enterprises that attract a large working-class population. The LGA is recognized a regional hub for education and healthcare due to the presence of major institutions like Ladoke Akintola University of Technology (LAUTECH) and two tertiary healthcare facilities, namely Bowen University Teaching Hospital and the LAUTECH Teaching Hospital. The majority of the population consists of Yoruba people, with both Christianity and Islam being widely practiced. These characteristics—high population density, economic engagement, and the presence of significant institutions—were vital in selecting Ogbomoso North as the location for the study.

### Study population

The study included caregivers in identifiable daycare centers in Ogbomoso North. Participants were identified through community engagement, and snowball sampling was employed due to the absence of a centralized registry (15, 16). A total of 136 participants enumerated. Only caregivers actively engaged in the Ogbomoso North daycare centers and those who provided consent were included in the study. Caregivers who were on leave, were sick, or refused to give their consent were excluded to maintain contextual relevance.

### Sample size and sampling technique

The sample size was calculated using the Yamane formula for finite populations. With a total caregiver population of 136, a confidence level of 95%, and a margin of error of 5%, the minimum required sample size was calculated to be 101 participants. To account for potential non-responses, an additional 10% was added, resulting in a final sample size of 111 participants. The Yamane formula can be adjusted for small populations, ensuring precision while avoiding oversampling. Using snowball sampling, caregivers were selected from all identified daycare centers in the Ogbomoso community. This sampling technique was employed due to the absence of a comprehensive registry of daycare centers in Ogbomoso North (15, 16).

The recruitment of participants occurred between February 2024 and April 2024, allowing the study to capture a current and accurate snapshot of the practice of infection prevention and control among daycare caregivers in the community.

### Study variables

#### Dependent variable

Infection prevention and control practices refer to how effectively caregivers implement methods to prevent and control infections in daycare settings. This variable is dependent because it is expected to change based on caregivers’ knowledge.

#### Independent variable

Caregivers’ knowledge refers to how much the caregivers know about infection prevention and control. It is considered independent because it is regarded as the cause of changes in the dependent variable.

### Study instrument

A self-structured questionnaire was used to collect data, which was constructed after a review of the literature. The questionnaire consisted of four sections: sociodemographic data of respondents, knowledge of infection prevention and control, practice of infection prevention and control, and factors associated with the practice of infection prevention and control.

After the initial development of the questionnaire, experts reviewed the draft to evaluate its content validity. Based on their feedback, several changes were made to improve clarity, relevance, and comprehensiveness. Specifically, ambiguous or confusing items were reworded for better understanding, redundant questions were removed, and additional items were added to address gaps identified by the experts. The revised questionnaire was subsequently pretested to ensure improved validity and reliability. The reliability coefficient was 0.96. To ensure data quality, the questionnaires were reviewed for completeness and consistency, and the data were double-entered and cross-verified.

The level of knowledge was assessed utilizing a 22-point scale. The responses were assigned codes of 1 for “no” and 2 for “yes,” except items 2 and 11, which were coded inversely. The criteria for “good” and “poor” knowledge were established by splitting the total achievable scores into two equal groups, consistent with a recognized method in a comparable study (17). Specifically, scores ranging from 1 to 10 were classified as poor practice, while those from 11 to 22 were considered good practice.

Additionally, infection prevention and control practices were assessed using a 72-point Likert scale. A score of 1 indicated “never,” 2 signified “rarely,” 3 represented “sometimes,” and 4 denoted” always.” The benchmarks for “good,” “moderate,” and “poor” practice were established by dividing the total possible scores into three equal segments, in line with the methodology used in a similar study (17). Specifically, scores ranging from 1 to 24 were classified as poor practice, scores ranging from 25 to 48 were classified as moderate practice, and scores ranging from 49 to 72 were classified as good practice.

### Data collection procedures

Data were collected using a structured self-administered questionnaire (paper-based) between February 2024 and April 2024 after ethical approval had been secured. Two undergraduate nursing students were recruited and trained effectively to assist the researcher. Respondents were approached at their respective daycare centers, and the objectives of the study, including its significance, were explained to them. The questionnaires were distributed only to caregivers who consented and were willing to participate in the study. The questionnaires were interpreted for those who only speak the indigenous language, and their right to anonymity and confidentiality was upheld.

### Statistical analysis

Data were coded and entered into the Statistical Package for the Social Sciences (SPSS) version 26.0, where cleaning and imputation procedures ensured data completeness and accuracy. Descriptive statistics were employed to summarize the key characteristics of participants, with frequency distribution tables and percentages used to depict sociodemographic details and describe the levels of knowledge and practice of infection prevention and control (IPC) among daycare caregivers. Furthermore, the relationships between caregivers’ IPC knowledge and practice and selected sociodemographic variables were examined using the chi-squared test. A two-tailed test was applied with statistical significance set at a *p*-value of less than 0.05.

### Ethical consideration

Formal ethical approval was obtained from the Oyo State Ministry of Health, with reference number AD 13/479/44695B, and permission to use the questionnaire was sought from the head of each daycare center. The respondents were informed about the study’s objectives and asked to provide their consent. Written informed consent was secured from each participant to ensure voluntariness and anonymity. They were informed that the questionnaire data results would be treated as highly confidential and anonymous. Questionnaires were distributed to the respondents by research assistants, who also provided interpretation for participants who did not understand English. The questionnaires were retrieved immediately to prevent any loss of data.

## Results

### Sociodemographic characteristics

Table 1 shows that the majority [66 (60%)] of the respondents were within the age group of 30–39 years and were from the Yoruba tribe [104 (94.5%)]. In terms of religion, the majority of them were Christians [94 (85.5%)], and they had completed tertiary education [68 (61.8%)]. In addition, many respondents [68 (61.8%)] received child care training, while a little above average had infection control training [62 (58.2%)].

**Table 1:** Demographic characteristics of daycare caregivers (*N* = 110).

Variables	Categories	Frequency	Percent (%)
Age	Less than 20 years	8	7.3
	20–29 years	22	20
	30–39 years	66	60
	40–49 years	14	12.7
Ethnicity	Yoruba	104	94.5
	Igbo	2	1.8
	Hausa	0	0
	Others	4	3.6
Religion	Christianity	94	85.5
	Islam	16	14.5
	Traditional	0	0
	Others	0	0
Years of work experience	Less than 5 years	71	63.6
	5–10 years	31	29.1
	11–15 years	4	3.6
	16 years and above	4	3.6
Level of education	Primary	19	9.1
	Secondary	32	29.1
	Tertiary	68	61.8
	None	0	0
Do you have child care training	Yes	68	61.8
	No	42	38.2
Do you have infection control training	Yes	62	58.2
	No	46	41.8

### Knowledge and practice of IPC among daycare caregivers

**Figure 1:**
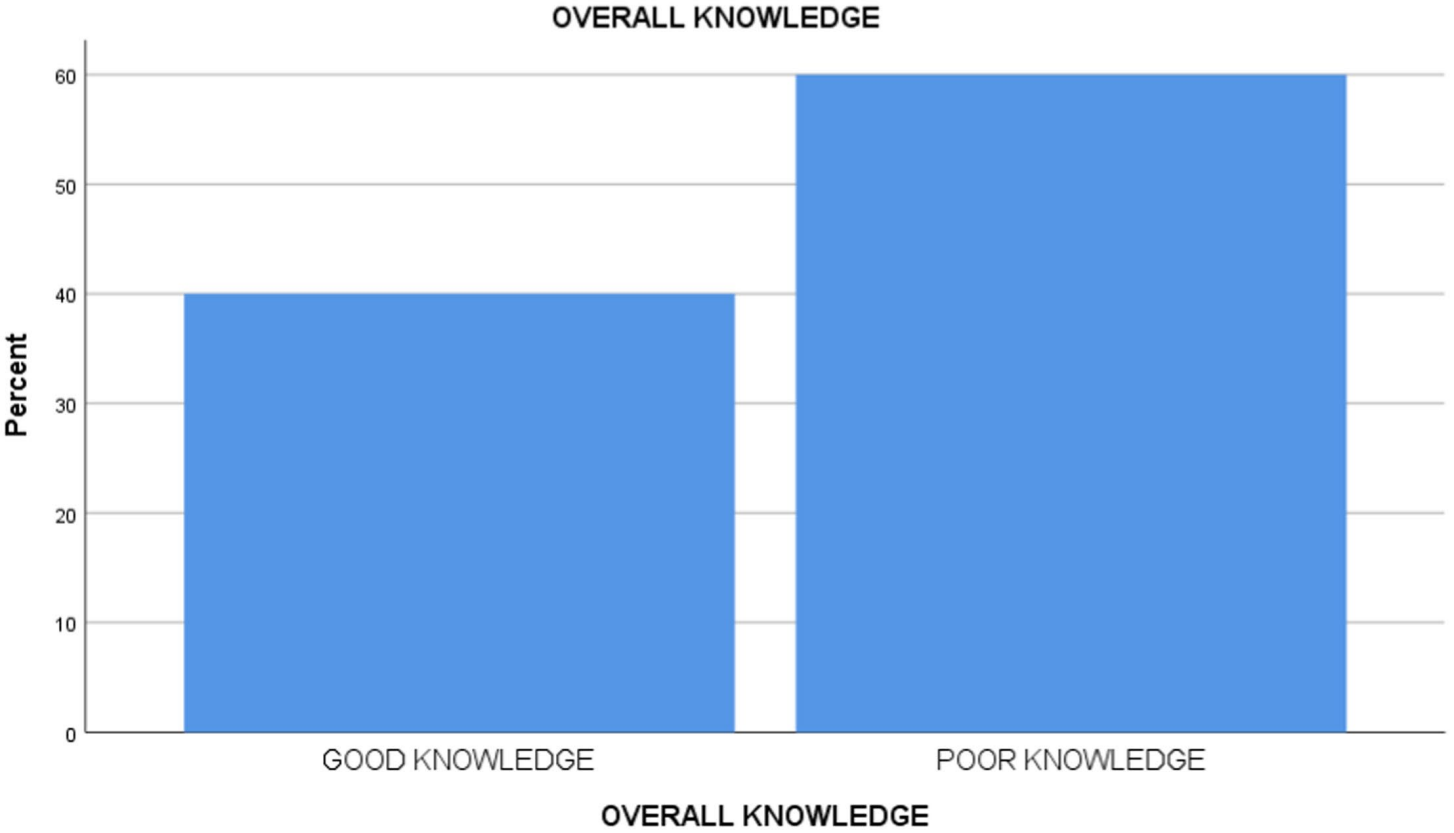
Level of knowledge.

Table 2 and Figure 1 reveals that 44 (40%) of the respondents had good knowledge of infection prevention and control practices, while 66 (60%) of them had poor knowledge of infection prevention and control practices. Furthermore, the study reveals that 36 (32.7%) of them had good practice of infection prevention and control, 22 (20%) had moderate practice, and 52 (47.3%) had poor practice (Table 3).

**Table 2:** Knowledge of infection prevention and control among daycare caregivers.

Variables	Categories	Frequency	Percent (%)
Infection prevention and control (IPC) is a practical, evidence-based approach that prevents children and daycare caregivers from being harmed by avoidable infections.	Yes	15	12.7
	No	95	87.3
Hand hygiene (washing hands with soap and water or using alcohol-based sanitizer) is the most essential way to prevent the spread of germs between children.	Yes	23	21.8
	No	87	78.2
Standard precautions are used to prevent the spread of all kinds of germs, not just from sick children.	Yes	27	23.6
	No	81	76.4
Handwashing should be carried out before and after playing with children, after wiping noses, and whenever your hands look dirty.	Yes	14	12.7
	No	96	87.3
Little coughs and sneezes can send germs to other people. We can help stop the spread by teaching people to cough or sneeze into their elbows.	Yes	25	21.8
	No	85	78.2
Children with cold or other infectious diseases are advised to stay at home to prevent the spread of diseases.	Yes	22	20
	No	88	80
Immunization is very important in infection control measures.	Yes	27	23.6
	No	83	76.4
Being in optimum health helps to prevent/ avoid infection transmission among daycare children.	Yes	24	21.8
	No	86	78.2
Wash hands after changing of diaper.	Yes	19	18.2
	No	91	81.8
Washing of hands before feeding.	Yes	18	16.4
	No	92	83.6
Exposing of children’s food.	Yes	40	36.4
	No	70	63.6
Overall knowledge		Good knowledge	Poor knowledge
		52 (47.3%)	58 (52.7%)

**Table 3:** Practice of infection prevention and control among daycare caregivers.

Variables	Never	Rarely	Sometimes	Always
I practice hand washing after using the toilet for each child	80 (72.7%)	20 (18.2%)	8 (7.3%)	2 (1.8%)
I wash my hands with only water	18 (16.4%)`	30 (27.3%)	44 (40%)	18 (16.4%)
I wash my hands with soap and water	72 (65.5%)	24 (21.8%)	8 (7.3%)	6 (5.5%)
Hand hygiene is practiced after I cough/sneeze/wipe my nose	50 (45.5%)	36 (32.7%)	22 (20%)	2 (1.8%)
After contact with urine, feces, and vomitus	80 (72.7%)	20 (18.2%)	8 (7.3%)	2 (1.8%)
I barely use alcohol hand sanitizer for hand hygiene	26 (23.6%)	42 (38.2%)	30 (27.3%)	12 (10.9%)
Cleaning of the toys is done daily	44 (40%)	34 (30.9%)	28 (25.5%)	4 (3.6%)
I use the children’s cloth to clean their nose	16 (14.5%)	24 (21.8%)	24 (21.8%)	46 (41.8%)
When cleaning vomitus, urine, and blood splashes, I wear personal protective equipment like gloves/apron	52 (47.3%)	30 (27.3%)	22 (20%)	6 (5.5%)
I pack the feeding utensils, such as plates, milk bottles, for the parent to wash	28 (25.5%)	32 (29.1%)	26 (23.6%)	24 (21.8%)
How often is the bathroom and toilet cleaned	34 (61.8%)	20 (18.2%)	18 (16.4%)	4 (3.6%)
The diaper is changed in the changing room	54 (49.1%)	24 (21.8%)	24 (21.8%)	8 (7.3%)
I separate sick children from the other children	56 (50.9%)	24 (21.8%)	24 (21.8%)	6 (5.5%)
How often do you clean the diaper changing room	56 (50.9%)	24 (21.8%)	24 (21.8%)	6 (5.5%)
Children who come to the school without cups share cups and water bottles with their friends	16 (14.5%)	24 (21.8%)	18 (16.4%)	52 (47.3%)
After changing a child’s diaper, the used diaper is wrapped in a leak-proof bag (polythene).	48 (43.6%)	24 (21.8%)	22 (20%)	8 (7.3%)
Immunization of the children is considered before admitting them to the center	44 (40%)	26 (23.6%)	34 (30.9%)	6 (5.5%)
Parent agree to have their sick child stay at home till they are well	48 (43.6%)	28 (25.5%)	30 (27.3%)	4 (3.6%)
Overall practice	Good practice	Moderate practice	Poor practice	
	46 (41.8%)	43 (39.1%)	21 (19.1%)	

### Association between knowledge and practice of IPC and sociodemographic variables

The study demonstrated that only the year of experience of caregivers has a *p*-value of 0.001; therefore, there is a significant relationship between years of work experience and infection prevention and control practices among daycare caregivers (see Table 4).

**Table 4:** Relationship between sociodemographic variables and infection prevention and control practices.

Variables		Practice			χ^2^	*df*	*p*-value
		Good practice	Moderate practice	Poor practice			
Age	<20 years	4	4	0	7.567	6	0.271
	20–29 years	6	2	14			
	30–39 years	20	16	30			
	40–49 years	6	0	8			
Level of education	Primary	6	2	2	4.331	4	0.363
	Secondary	14	4	14			
	Tertiary	16	16	36			
Religion	Christianity	28	20	46	1.306	2	0.521
	Islam	8	2	6			
	Others	0	0	0			
Ethnicity	Yoruba	36	20	48	2.759	4	0.599
	Igbo	0	0	2			
Years of experience	<5 years	20	18	32	**10.523**	**6**	**0.001**
	5–10 years	8	4	20			
	11–15 years	4	0	0			
	≥16 years	4	0	0			

*p* value is significant < 0.05.

### Association between knowledge and practice of IPC among daycare caregivers

There is a significant relationship between the knowledge of IPC and the practice of infection prevention and control among daycare caregivers in the Ogbomoso community (*p* < 0.05) (Table 5).

**Table 5:** Relationship between knowledge of IPC and practice of infection prevention and control among daycare caregivers.

Variable		Practice			*χ* ^2^	*df*	*p*-value
		Below average	Average	Above average			
Knowledge	Good	28 (66.7%)	8 (19%)	6 (14.3%)	20.281a	4	**0.000**
	Poor	8 (12.1%)	14 (21.2%)	44 (66.7%)			

*p* value is significant < 0.05.

### Discussion of findings

This study found that a significant number of respondents were in the age group of 30–9 years, suggesting that they belong to a relatively young population. This demographic is likely to possess not only strength but also the proactive disposition required to raise and look after children efficiently. These findings are in agreement with previous studies, such as those by Obiagwu and Ajayi (12) and Atekoja et al. (18), which reported the mean caregiver age of 35.3 and 33.7 years, respectively. Surprisingly, it was discovered that approximately two-thirds of the participants had received some form of childcare training; however, as noted, this level of training did not lead to any improvement in knowledge or in the actual implementation of infection prevention and control practices among daycare children. In sharp contrast, a similar study revealed that as many as 74.3% of their respondents received no pre-employment childcare-related training (12).

Findings from this study clearly showed that more than half of the daycare caregivers had a significant lack of knowledge and understanding regarding the critical domain of infection prevention and control measures. This level of expertise is alarming and inadequate; therefore, it might be one of the contributing factors that could adversely influence the quality and effectiveness of the real-world practice of infection prevention and control among caregivers entrusted with the well-being of children. This finding is further supported by numerous studies that have similarly reported alarmingly low levels of knowledge in infection prevention and control measures among caregivers in daycare centers in different settings (5, 19). Notably, some of these studies reported findings quite contrary to what has been described above. It was recorded that both the private and public educators within day care centers had a good understanding and accurate knowledge of the fundamentals of infection control (20).

**Figure 2:**
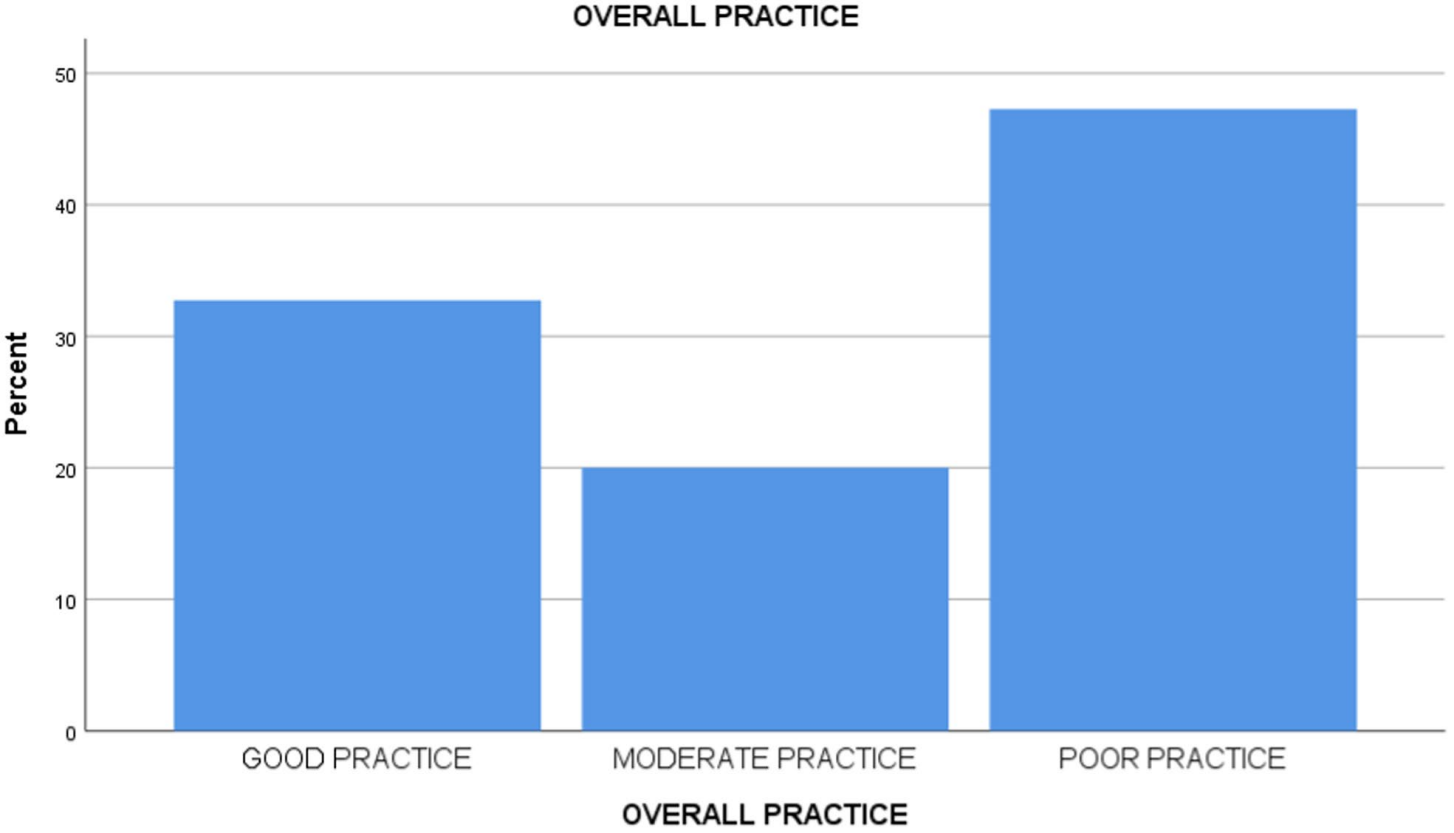
Level of practice.

More precisely, while investigating the level of practice of infection prevention and control by the caregivers working in daycare centers, this study discovered the identification of an extremely low level of implementation of infection prevention and control practices, as evidenced by the fact that the subjects of this research performed strikingly below the average (Figure 2). This may not be surprising, considering that the level of their knowledge is actually related to their real practice of infection prevention and control strategies. Moreover, the inadequacy noted could also have emanated from an inability of the participants to transform theoretical knowledge imparted in training into actual, practical fieldwork. In the same light, the study by Tahoun et al. (5) also supported the present study’s findings in demonstrating a similarly low level of practical engagement with infection prevention and control measures among the relevant population. However, Schmidt et al. (21) reported a statistically higher rate of compliance with infection prevention and control practices, thereby indicating notable differences in results between studies.

This study found that the length of time caregivers had been in practice was positively correlated with compliance with the practice of infection prevention and control measures. This would imply that the longer the period of professional experience, the more the daycare caregivers would comply with set protocols and practices for preventing the transmission of infections; hence increasing the standards of safety and health in childcare centers. The fact that years of experience among daycare workers significantly correlate with the practice of infection prevention and control is supported by literature that considers caregivers’ level of knowledge and experiences about infection control. For example, Tahoun et al. (5) indicate that knowledge of infectious diseases among caregivers is an influential factor in their practice, infection control-wise; only a few percent have demonstrated adequate knowledge. Similarly, Kang and Kim (22) also found that experiences and knowledge play an essential role in shaping infection control practices of childcare teachers. More importantly, Ismail et al. (19) demonstrated that education interventions could enhance the practice of caregivers of under-5 children; this suggests that, other than experience, good infection management practices also need training. Generally, these studies collectively support the idea that experiences and education go together in improving infection prevention methods in childcare (22, 23).

Furthermore, results from this research have indicated a high and significant association between the knowledge acquired by child daycare workers about the practice of infection prevention and control, and eventual infection prevention and control adopted among the children within these daycare centers. The finding provides a basis for emphasizing the importance of the caregivers’ knowledge level in determining the effectiveness of their practical applications in infection prevention. This logically suggests that one who lacks sufficient knowledge on any particular subject will not meet expectations when putting that knowledge to practical use. These findings, therefore, agree with the literature on the statistical association between knowledge of IPC and its practice among daycare caregivers, placing caregivers’ knowledge at the center of implementing effective health practices. Empirical studies, for example, find that caregivers in daycare settings often demonstrate poor knowledge of IPC and that this is indeed related to poor practices; for instance, one study found that only 2.5% of caregivers showed adequate knowledge, and just 17% had positive practice scores (5). In this line of thought, a study noted that as much as caregivers possessed knowledge on health practices, this did not imply the proper translation into application, as evident in the low levels of correlation established between knowledge and practice of oral health care by the same (24). Most of the studies further stress the issue of focused education, and that increasing caregivers’ knowledge markedly improves their health practices, which may affect the health outcome among children (12, 25). Therefore, this study recommends that adequate training on the practice of infection prevention and control will improve the level of knowledge and practice of infection prevention and control.

### Implications of the findings

In daycare settings, these results reveal a critical vulnerability: insufficient IPC practices can create a conducive environment for the spread of infections among young children, who are inherently more susceptible to infectious diseases. Given that daycare centers serve as a primary point of care for working-class families, the lack of robust IPC measures not only jeopardizes the health of children but also potentially facilitates the transmission of infections in the broader community. This necessitates implementing routine, in-depth IPC training programs, ongoing supervision, and allocating sufficient resources to ensure that standard IPC protocols are consistently followed (5, 18, 26).

On a national policy level, the findings call for a review and strengthening of regulations governing childcare centers. Policymakers should consider mandating comprehensive IPC training for all daycare caregivers, regardless of their years of experience, as the study indicates that even experienced personnel may exhibit deficiencies in practice (18). Furthermore, conducting regular audits and inspections of daycare centers can help ensure adherence to established IPC guidelines, thereby reducing the likelihood of infection outbreaks. These policy interventions are critical for safeguarding public health within childcare settings and preventing broader public health crises that could result from uncontrolled transmission of infectious diseases.

### Limitations of the study

The study is based on self-reported responses from daycare caregivers without direct observation, which may introduce bias from social desirability or inaccurate recall. Additionally, since the study is cross-sectional, it captures data at a single point, preventing the analysis of changes in knowledge and practice over time.

## Conclusion

This current study concludes that infection prevention and control measures are integral to preventing communicable diseases among daycare caregivers. This study discovered that fewer than average daycare caregivers had good knowledge of infection prevention and control. Similarly, fewer than average daycare caregivers had good practice of infection prevention and control. The main associated factors of inadequate practice of IPC among the daycare caregivers include fewer years of experience of caregivers and knowledge of caregivers about IPC. Thus, the study suggests that engagement of daycare caregivers to improve their level of understanding of infection prevention and control, including its benefits and practices, should be encouraged to reduce the risk of infectious agents being transmitted by daycare caregivers.

## Data Availability

The raw data supporting the conclusions of this article will be made available by the corresponding author upon reasonable request.

## References

[1] Pidjadee C Soh KL Attharos T Soh KG. The effect of infection prevention and control programme for childcare workers in daycare centres: a systematic review. J Pediatr Nurs. (2024) 79:116–25. doi: 10.1016/j.pedn.2024.09.002, PMID: 39255691

[2] De Hoog ML Venekamp RP van der Ent CK Schilder A Sanders EA Damoiseaux RA. Impact of early daycare on healthcare resource use related to upper respiratory tract infections during childhood: prospective WHISTLER cohort study. BMC Med. (2014) 12:1–8. doi: 10.1186/1741-7015-12-107 PMC4098954 24965189

[3] McGrath BJ. Identifying health and safety risks for childcare workers. AAOHN J. (2007) 55:321–5. doi: 10.1177/216507990705500804, PMID: 17847626

[4] Uhari M Möttönen M. An open randomized controlled trial of infection prevention in child day-care centers. Pediatr Infect Dis J. (1999) 18:672–7. doi: 10.1097/00006454-199908000-00004, PMID: 10462334

[5] Tahoun MM Hasab AAH El-Nimr NA. Infection control in child daycare centers: logistics, knowledge, and practices of caregivers. J Egypt Public Health Assoc. (2019) 94:16–7. doi: 10.1186/s42506-019-0016-7, PMID: 32813102 PMC7364692

[6] Kilpatrick M Hutchinson A Manias E Bouchoucha SL. Paediatric nurses’, children's and parents’ adherence to infection prevention and control and knowledge of antimicrobial stewardship: a systematic review. Am J Infect Control. (2021) 49:622–39. doi: 10.1016/j.ajic.2020.11.025 33285224

[7] Oriekwo JO Williams JO Peekate LP. Bacterial population of environmental surfaces in child-care centres in Omoku and Port Harcourt, Nigeria. J Adv Microbiol Res. (2023) 4:53–9. doi: 10.22271/micro.2023.v4.i2a.101

[8] Wilkie ED Alao JO Sotala TT Oluduro AO. Molecular characterisation of virulence genes in bacterial pathogens from daycare centres in Ile-Ife, Nigeria: implications for infection control. BMC Infect Dis. (2024) 24:1196. doi: 10.1186/s12879-024-10095-8, PMID: 39443869 PMC11515781

[9] Wilkie ED Alao JO Thonda OA Oluduro AO. Prevalence, distribution, and antibiotic resistance of bacterial isolates in daycare centers in Ile-Ife, Nigeria. Antimicrob Steward Healthc Epidemiol. (2024) 4:e220. doi: 10.1017/ash.2024.473, PMID: 39758874 PMC11696597

[10] Adede ME Barde AJ Eniola BM Hassan MT Aanuoluwapo OA. Public health challenges and solutions in urban areas of Nigeria. Int J Health Pharm Res. (2024) 9:39–52. doi: 10.56201/ijhpr.v9.no3.2024.pg39.52

[11] Ackerman SJ Duff SB Dennehy PH Mafilios MS Krilov LR. Economic impact of an infection control education program in a specialized preschool setting. Pediatrics. (2001) 108:e102–2. doi: 10.1542/peds.108.6.e102, PMID: 11731629

[12] Obiagwu AE Ajayi IO. Disease prevention: childcare practices of caregivers in day-cares/pre-schools in Ibadan, Nigeria. Int J Community Med Public Health. (2021) 9:16. doi: 10.18203/2394-6040.ijcmph20214975

[13] Asekun-Olarinmoye E Oyemade A Lawoyin T. Health status of children aged under two years cared for in day-care centres and the home environment in Ibadan, Nigeria. J Community Med Prim Health Care. (2005) 17:33–7. doi: 10.4314/jcmphc.v17i1.32423

[14] Ejemot-Nwadiaro RI Ehiri JE Arikpo D Meremikwu MM Critchley JA. Hand-washing promotion for preventing diarrhoea. Cochrane Database Syst Rev. (2021) 12:265. doi: 10.1002/14651858.CD004265.pub4 PMC8094449 33539552

[15] Biernacki P Waldorf D. Snowball sampling: problems and techniques in chain referral sampling. Sociol Methods Res. (1981) 10:141–63. doi: 10.1177/004912418101000205

[16] Sadler GR Lee HC Lim RS Fullerton J. Recruitment of hard-to-reach population subgroups via adaptations of the snowball sampling strategy. Nurs Health Sci. (2010) 12:369–74. doi: 10.1111/j.1442-2018.2010.00541.x, PMID: 20727089 PMC3222300

[17] Radhakrishnan R Datchanamoorthy M Narayanasamy D Leela KV. Assessment on infection prevention and control knowledge among medical professionals in south Indian population. J Infect Dev Ctries. (2023) 17:468–76. doi: 10.3855/jidc.17377 37159897

[18] Atekoja O Kio J Ogundare T Ajiboye R. Availability of infection prevention measures and occurrence of infections in childcare centres in Sagamu local government area, Ogun state, Nigeria. Ann Health Res. (2023) 9:146–57. doi: 10.30442/ahr.0902-07-200

[19] Ismail FH Osman M El-Amin EO. Effect of an interventional health education program on the practice of caregivers towards infection control measures in Mygoma orphanage center 2014-2017. Saudi J Nurs Health Care. (2020) 3:6. doi: 10.36348/sjnhc.2020.v03i12.006

[20] Al-Shatari SAH Ghafouri TS. Knowledge, attitude, and practice of nurseries' workers toward infection prevention among the children. Al-Kindy Coll Med J. (2021) 17:168–74. doi: 10.47723/kcmj.v17i3.297

[21] Schmidt W-P Wloch C Biran A Curtis V Mangtani P. Formative research on the feasibility of hygiene interventions for influenza control in UK primary schools. BMC Public Health. (2009) 9:1–8. doi: 10.1186/1471-2458-9-390 19832971 PMC2770489

[22] Kang K-S Kim E-Y. Factors influencing performance about practice of infection management by child care center teachers about respiratory tract infections. J Digit Converg. (2017) 15:315–26. doi: 10.14400/JDC.2017.15.10.315

[23] Blancarte-Fuentes E Álvarez-Aguirre A Tolentino-Ferrel MR. Primary caregiver, transmitting agent of healthcare-associated infections: literature review. SANUS Rev Enferm. (2019) 4:34–50. doi: 10.36789/sanus.vi12.140

[24] Halim A Yusof RM Abdullah SN. The correlation between oral health knowledge & attitude towards practice of caretakers in day-care centres. IIUM Med J Malaysia. (2018) 17:189–94. doi: 10.31436/imjm.v17i2.992

[25] Aboubakr RM Ramadan RI. Knowledge and practice of caregivers about prevention of early childhood caries. Egypt Dent J. (2019) 65:1727. doi: 10.21608/EDJ.2015.71727

[26] Andrup L Krogfelt KA Stephansen L Hansen KS Graversen BK Wolkoff P. Reduction of acute respiratory infections in day-care by non-pharmaceutical interventions: a narrative review. Front Public Health. (2024) 12:1332078. doi: 10.3389/fpubh.2024.1332078 38420031 PMC10899481

